# Does pronounceability modulate the letter string deficit of children with dyslexia? A study with the rate and amount model

**DOI:** 10.3389/fpsyg.2014.01353

**Published:** 2014-12-02

**Authors:** Chiara V. Marinelli, Daniela Traficante, Pierluigi Zoccolotti

**Affiliations:** ^1^Neuropsychology Research Centre, IRCCS Santa LuciaRome, Italy; ^2^Department of Psychology, Catholic University of MilanMilan, Italy; ^3^NeuroMI – Milan Center for NeuroscienceMilan, Italy; ^4^Department of Psychology, Sapienza University of RomeRome, Italy

**Keywords:** developmental dyslexia, lexical decision, Reicher–Wheeler paradigm, pronounceability, global factor, letter string

## Abstract

The locus of the deficit of children with dyslexia in dealing with strings of letters may be a deficit at a pre-lexical graphemic level or an inability to bind orthographic and phonological information. We evaluate these alternative hypotheses in two experiments by examining the role of stimulus pronounceability in a lexical decision task (LDT) and in a forced-choice letter discrimination task (Reicher–Wheeler paradigm). Seventeen fourth grade children with dyslexia and 24 peer control readers participated to two experiments. In the LDT children were presented with high-, low-frequency words, pronounceable pseudowords (such as DASU) and unpronounceable non-words (such as RNGM) of 4-, 5-, or 6- letters. No sign of group by pronounceability interaction was found when over-additivity was taken into account. Children with dyslexia were impaired when they had to process strings, not only of pronounceable stimuli but also of unpronounceable stimuli, a deficit well accounted for by a single global factor. Complementary results were obtained with the Reicher–Wheeler paradigm: both groups of children gained in accuracy in letter discrimination in the context of pronounceable primes (words and pseudowords) compared to unpronounceable primes (non-words). No global factor was detected in this task which requires the discrimination between a target letter and a competitor but does not involve simultaneous letter string processing. Overall, children with dyslexia show a selective difficulty in simultaneously processing a letter string as a whole, independent of its pronounceability; however, when the task involves isolated letter processing, also these children can make use of the ortho-phono-tactic information derived from a previously seen letter string. This pattern of findings is in keeping with the idea that an impairment in pre-lexical graphemic analysis may be a core deficit in developmental dyslexia.

## INTRODUCTION

In lexical decision tasks (LDTs) participants are required to discriminate between real words and foils. The difficulty of the discrimination varies according to the characteristics of the foils: as orthographic and phonological overlap between words and foils increases, the LDT becomes progressively more difficult. Participants are faster and more accurate at rejecting unpronounceable illegal non-words (i.e., letter strings such as GLDT) than pronounceable pseudowords (i.e., nonsense strings of letters that respect the orthographic rules of a given language but have no semantic content such as RINAFO; e.g., Holcomb and Neville, 1990; Forster et al., 2003; Ratcliff et al., 2004). Evans et al. (2012) reported that, as foils become increasingly word-like, non-word (“no”) responses became significantly slower and less accurate: reaction times (RTs) were shorter, and accuracy was higher, for consonant strings compared to pseudowords. Moreover, also real word (“yes”) responses in the context of increasingly word-like foils were slower and less accurate. Passing from non-word to pseudoword foils there is a progressive increase in pronounceability as well as in orthographic and phonological similarity to real words. Moreover, as foils become more word-like and orthographic and phonological overlap increases, foils produce more activation of similar words (Harm and Seidenberg, 2004) and the discrimination becomes more difficult, indicating that a higher level of activation is required for a real word (“yes”) decision to avoid false alarms.

Some authors investigated whether pronounceability influences the depth of processing required for lexical decision. James (1975) found that this task involves retrieval of semantic information (as highlighted by the concreteness effect) only in the presence of pronounceable distractors, while, decreasing the similarity between words and foils, the use of unpronounceable distractors makes the semantic retrieval unnecessary. Similarly, Evans et al. (2012) reported smaller effects of imageability and semantic priming as decision difficulty and RTs decreased from pseudo-homophone, to pseudoword, and non-word foil contexts, with semantic effects minimized with unpronounceable foils. Moreover, semantic effects increased significantly as decisions became harder (and slower) with more word-like foils. Dimitropoulou et al. (2010) examined the masked onset priming effect (MOPE) by manipulating the primes’ lexicality, frequency, and pronounceability. The MOPE indicates faster naming latencies when a target word (e.g., BREAK) is preceded by a briefly presented masked prime that shares its initial sound with the target (e.g., belly) compared to when it does not (e.g., merry) or when it rhymes with it (e.g., stake; Forster and Davis, 1991). This effect has been interpreted as an advantage in speech planning of the response or as evidence of prime processing by the non-lexical route. Dimitropoulou et al. (2010) found the MOPE for all types of stimuli but unpronounceable non-words, a result favoring the speech planning hypothesis. Finally, pronounceability has been examined also in terms of the facilitation present for repeated stimuli. In a LDT, the repetition priming was larger in experiments with pseudowords than in experiments with non-words (Ratcliff et al., 2004).

The effect of pronounceability has been evaluated not only in LDTs, but also with other experimental paradigms. Seidenberg et al. (submitted) examined whether pronounceabililty influences the reading aloud process. Participants had greater difficulty in naming non-words containing grapho-tactically illegal sequences of letters (e.g., JULBZ) as compared to grapho-tactically legal non-words containing digraphs (i.e., multi-letter graphemes that map onto a single phoneme, e.g., the “ee” in NEESH). These findings indicate that pronounceability is a key factor in determining naming latencies, with pronounceable pseudowords being responded to faster than unpronounceable non-words. Differences are also observed when participants have only to identify a single letter in the stimulus in a post-cued letter-in-string identification task. Thus, letter recognition is more accurate in the context of a pronounceable pseudoword than in the context of a consonant string, the so-called pseudoword superiority effect (PSE; e.g., Baron and Thurston, 1973; Spoehr and Smith, 1975; Grainger and Jacobs, 1994, 2005).

It is worth noting that when also reading proficiency is taken into account the framework becomes more complex. In fact, if two groups (e.g., dyslexic and proficient readers) vary in general speed of processing (hereafter referred to as the global factor), group differences in latencies would depend on both the difficulty of a given task and the general group differences in processing speed (Faust et al., 1999). Then, for groups showing global differences in performance, one should expect to find over-additivity effects; i.e., the absolute group differences in performance would tend to grow as a function of task difficulty over and above the characteristics of the specific experimental manipulations (Faust et al., 1999). The presence of over-additivity may induce overestimation or underestimation of the contribution of specific variables modulating reading performance. Therefore, it may not be easy to identify the presence of a deficit in processing unpronounceable stimuli because of differences in task difficulty across experimental conditions and their interaction with basic group differences in rate of information processing. Models such as the rate and amount model (RAM; Faust et al., 1999) reveal the presence and characteristics of the global factor in information processing by distinguishing between the performance of dyslexic and proficient readers and isolating the conditions in which children with dyslexia show specific deficits not ascribable to over-additivity (Zoccolotti et al., 2008).

In previous studies, we found that a single global factor accounted for a very large proportion of the impaired performance of children with dyslexia in making lexical decisions and reading words and pseudowords (Di Filippo et al., 2006; Zoccolotti et al., 2008; Marinelli et al., 2011). Other studies showed that the global factor was present for orthographic but not pictorial stimuli (Zoccolotti et al., 2008) and in the visual, but not the auditory, modality (Marinelli et al., 2011). The global factor did not emerge in a variety of letter and bigram tasks even though their general difficulty was made similar to that of letter strings (i.e., both words or pseudowords; De Luca et al., 2010). In fact, tasks mapping letter (and bigram) recognition loaded on a separate factor other than that accounting for words and non-words.

These studies indicate that children with dyslexia are selectively impaired in processing visually presented strings of letters with or without lexical value. We have proposed that this deficit has a pre-lexical graphemic locus (e.g., De Luca et al., 2010), i.e., marks an impairment in forming a graphemic description of the letter string (Zoccolotti et al., 2008). This idea is in keeping with other proposals based on imaging and lesional studies of the so-called “visual word form area” (VWFA; Cohen et al., 2000, 2002). The local combination detector (LCD) model (Dehaene et al., 2005) posits that written words are encoded by a hierarchy of detectors tuned to increasingly larger and more complex word fragments (visual features, single letters, bigrams, quadrigrams and, possibly, words). At the neural level, information from letter features and single letter converges on the VWFA; here, a posterior-to-anterior gradient is present with a progression in selectivity to increasingly word-like stimuli (e.g., Dehaene et al., 2004; Vinckier et al., 2007). Over years of practice, frequent combinations of letters are selected to be represented by dedicated neurons (Cohen et al., 2008), and the VWFA becomes attuned to the regularities of the writing system, yielding fast parallel processing in reading (Vinckier et al., 2007; Cohen et al., 2008). Importantly, several studies found that dyslexic individuals show selective hypo-activation of the VWFA (for a review see Richlan et al., 2009). In a similar vein, Marsh and Hillis (2005) proposed that this area is involved in the computation of a prelexical “grapheme description” independent of case, font, location, or orientation. Notably, such graphemic description does not require stored knowledge of spelling or spelling-sound correspondences. In this perspective, the reading impairment of children with dyslexia might be ascribed to a deficit at the level of graphemic analysis.

Another interpretation of the dyslexic deficit is that the impairment is related to the inability to bind orthographic and phonological information (Ziegler et al., 2010; van den Broeck and Geudens, 2012). Evidence in this direction comes from imaging studies indicating a close association between letter and speech sounds early in development (for a review see Blomert, 2011). Accordingly, effective letter–speech sound integration is an emergent property of learning to read supported by an interrelated network of visual, auditory, and heteromodal brain areas. There is evidence that dyslexic individuals are impaired in letter–speech sound integration. For example, in a functional magnetic resonance imaging (fMRI) study, Blau et al. (2009) reported that adult dyslexic readers showed underactivation of the superior temporal cortex for the integration of letters and speech sounds. In a further study, Blau et al. (2010) reported that, unlike control readers, cortical responses to speech sounds of dyslexic individuals were not modulated by letter–speech sound congruency. In a complementary line of research, it has been reported that proficient readers activate orthographic representations in phonological LDT while children with dyslexia do not (van der Mark et al., 2011). Thus, dyslexic individuals fail to activate orthographic representations during spoken language processing. Finally, also previously described results on the VWFA are not necessarily incompatible with an orthographic–phonological binding perspective. The presence of interactions between orthographic and phonological processing is suggested by evidence indicating connections between the VWFA and language areas (Cai et al., 2008; Greve et al., 2013). First, it has been noted that the VWFA shows a clear lateralization with only the left, linguistic, hemisphere that becomes specialized for reading (Cai et al., 2008); furthermore, it has been reported that asymmetries in the VWFA are correlated with the ear advantage in a dichotic listening task (Greve et al., 2013). Overall, it has been proposed that a deficit in orthographic–phonological binding may represent a proximal cause of the reading slowness in dyslexia and may also help understanding the deficit in reading fluency of these individuals (Blomert, 2011).

In the present research, we tested these alternative hypotheses by evaluating the role of stimulus pronounceability on accuracy and latency in two different tasks: a LDT (Experiment 1), and a two-alternative forced-choice task (the so-called Reicher–Wheeler paradigm: see Reicher, 1969; Wheeler, 1970; Experiment 2).

In the LDT we are interested in assessing whether the impairment shown by children with dyslexia in processing letter strings is present only for pronounceable words and pseudowords or it is also detectable (and of a similar size) with unpronounceable letter strings. The first outcome would favor an orthographic–phonological binding interpretation while the latter a pre-lexical graphemic locus of the reading deficit. In fact, strings of consonants are not pronounceable and, as such, would not activate orthographic–phonological binding to the same extent as words or pseudowords. Therefore, based on the orthographic–phonological binding hypothesis we would expect a smaller deficit for non-words as compared to words and pseudowords (once the effect of over-additivity is controlled for). Based on the hypothesis of a letter string graphemic deficit, no interaction between groups and item pronounceability would be expected. All condition means in the LDT (for words, pseudowords, and non-words) are supposed to fit, in the RAM, with the same letter string factor.

The Reicher–Wheeler paradigm proposes the same types of stimuli used in the LDT, i.e., words, pseudowords and non-words, but does not require the simultaneous processing of several letters. In fact, the decision is to be made on the discrimination between a target letter and a competitor in the context of a previously displayed letter string. Assuming that the main impairment of children with dyslexia has to do with simultaneous letter string processing and not with single letters, the lack of a global factor in the RAM model might be expected. However, examining the context effects (i.e., the lexicality and the pronounciability of the prime) it is possible to test whether children with dyslexia can gain advantage from lexical activation and pronounceability in graphemic processing as much as control children.

In particular, testing for the PSE (i.e., letter identification is more accurate in the context of a pseudoword than in the context of a unpronounceable non-word) would allow evaluating whether children can take advantage from a pronounceable letter string which forms a typical orthographic context in Italian. Furthermore, testing for the word superiority effect (WSE; i.e., letter identification is more accurate in the context of a word than in the context of a pronounceable pseudoword) would allow evaluating whether children can take advantage of the lexical activation triggered by a word context in the successive letter recognition. According to the orthographic–phonological binding hypothesis we would expect, for children with dyslexia, a lack of both PSE and WSE, while for skilled readers a role of lexicality and pronounceability of the context is expected. On the other hand, the letter string graphemic deficit hypothesis would predict no differences in the effect size of the WSE and PSE in relation to reading proficiency, as the task does not require any decision on the lexicality of a specific letter combination, but only the discrimination between a single target letter and a competitor one. Furthermore, as no selective deficit in orthographic–phonological interaction is envisaged, one would expect children with dyslexia to be able to take advantage of the ortho-phonotactic regularities of the language (i.e., they are expected to show a PSE).

In order to apply the RAM model to the data from both the LDT and Reicher–Wheeler paradigms, we examined the speed of processing of children with dyslexia in responding to these tasks. There is evidence that, even though most studies on the Reicher–Wheeler paradigm focused on accuracy, parallel effects have also been reported with time measures (RTs and visual evoked potentials; Ziegler et al., 1997; Martin et al., 2006). To test the global factor it is important to have a sizeable spread of performances across conditions (Faust et al., 1999). To this aim, in LDT we presented high-frequency words, low-frequency words, pseudowords and non-words, varying for length within each category (from 4 to 6 letters), for a total of 12 different conditions. Previous data on Italian children indicate that children with dyslexia show frequency (e.g., Barca et al., 2006) and lexicality (e.g., Zoccolotti et al., 2008) effects both in reading and LDT (Paizi et al., 2013). These effects tend to be greater in children with dyslexia than in typically developing children in raw data analyses but, typically, this group interaction disappears when over-additivity is controlled for (Di Filippo et al., 2006; Zoccolotti et al., 2008; Paizi et al., 2013). In the Reicher–Wheeler paradigm, primes were 4-letter high-frequency words, pseudowords and non-words, with the target letter in first, second, or third position, for a total of nine different conditions. Note that no data are yet available on this paradigm on Italian children.

## EXPERIMENT 1

The first experiment examined the performance of children with dyslexia and typically developing readers in a LDT with non-words, pseudowords, low-frequency words, and high-frequency words presented intermixed.

### METHOD

#### Participants

Participants were 41 fourth grade children with a normal intelligence (according to the Raven’s Coloured Progressive Matrices; Pruneti et al., 1996) and adequate socio-educational conditions. In particular, there were 17 children with dyslexia (10 Male and 7 Female; mean age = 9.50 years, *SD* = 0.30) and 24 control readers (9 Male and 15 Female; mean age = 9.50 year, *SD* = 0.30). Children were selected by our psychology unit during a screening for learning disabilities carried out in local public schools of Rome. Parents were informed of the screening procedure and authorized their child’s participation.

Children with dyslexia were selected for a marked reading delay (at least 2 *SD*s below normative data) in accuracy and/or speed in reading a text passage (MT reading test, Cornoldi and Colpo, 1998). None of the children had received treatment for their reading impairment. Criteria for inclusion in the control group included normal reading speed and accuracy on the MT reading test (Cornoldi and Colpo, 1998). Control participants were comparable to children with dyslexia for sex (χ^2^ = 1.06, n.s.), age (*t*_(40)_ = 0.08, n.s.), and Raven’s test performance (*F*_(1,41)_ = 0.04, n.s.). On the MT reading test (Cornoldi and Colpo, 1998), mean *z* scores of control participants were near zero for all parameters (accuracy: –0.03, *SD* = 0.80; speed: –0.10, *SD* = 0.57). By contrast, children with dyslexia performed worse than control readers in reading accuracy (mean *z* score = –3.00, *SD* = 1.20; *t*_(40)_ = 9.45, *p* < 0.0001) and speed (mean *z* score = –1.75, *SD* = 1.25; *t*_(40)_ = 5.71, *p* < 0.0001). As a group, children with dyslexia showed only mildly defective performance in reading comprehension (mean *z* score = –0.27, *SD* = 0.43), but they scored lower than the control readers (mean *z* score = 0.04; *SD* = 0.26; *t*_(40)_ = 2.84, *p* < 0.01).

A number of other tests were used to qualify the reading and cognitive profile of children with dyslexia. **Table 1** reports the performance on the *Word and Non-word Reading Test* (Zoccolotti et al., 2005), a standard test of words and non-words reading (for basic characteristics of this and other tests please refer to the legends of the Tables). As it can be seen from the table, performance of the group of children with dyslexia was significantly lower than that of control readers across all conditions. The table reports also the proportion of children performing at least 2 *SD*s below normative data; across all conditions 16 children out 17 children showed a deficit in at least one subset of the test (the odd one out had a moderate impairment in this test, about –1 *SD*, across all conditions). Impairment appeared more marked for low-frequency (both short and long) words and long high-frequency words.

**Table 1:** Performance of children with dyslexia and control readers in the *Word and Non-word Reading Test* (Zoccolotti et al., 2005).

	ACCURACY							SPEED						
	Control readers		Children with dyslexia					Control readers		Children withdyslexia				
	*M*	*SD*	*M*	*SD*	% path. perf.	*t*_(40)_	*p*	*M*	*SD*	*M*	*SD*	% path. perf.	*t*_(40)_	*p*
Short HF words	0.36	0.41	-0.66	0.94	23.53	4.7	< 0.001	0.7	1.67	-1.36	1.06	52.94	4.46	< 0.001
Long HF words	0.42	0.75	-2	1.75	64.71	6.03	< 0.001	0.65	0.95	-2.48	1.26	88.24	9.01	< 0.001
Short LF words	0.36	0.63	-1.42	1.18	47.06	6.22	< 0.001	0.6	1.21	-2.3	1.47	58.82	6.89	< 0.001
Long LF words	0.25	0.84	-1.52	1.32	47.06	5.21	< 0.001	0.8	1.08	-1.91	1.18	70.59	7.57	< 0.001
Short pseudo-words	0.29	0.48	-1.03	1.17	35.29	4.96	< 0.001	0.17	1.33	-1.23	1.11	41.18	3.55	< 0.01
Long pseudo-words	0.29	0.97	-1.36	1.52	35.29	4.22	< 0.001	0.48	1.13	-1.45	1.36	47.06	4.93	< 0.001

*Values indicate *z* scores as compared to normative values (negative values indicate lower performance). Path. perf., pathological performance; HF, high-frequency; LF, low-frequency. The test is made of six A4 sheets, one for each subset of stimuli. There are 30 stimuli in each subset. Short stimuli are 4-, 5-letter long, while long stimuli are 8-, 10-letter long. Pseudo-words were generated from high-frequency words.*

On the *Test for the Diagnosis of Orthographic Deficit in Childhood* (Angelelli et al., 2008; **Table 2**), children with dyslexia, as a group, showed impaired performance on all subtests, except for that on spelling regular words. About half of the children with dyslexia showed severely impaired performance in spelling. **Table 3** reports the performance of the two groups of children on phonological and visual attention span tests (for information on these tests please refer to the Table legend). As a group children with dyslexia showed lower performance than control readers in several of these tests, i.e., the *Visual Attention Span* (Bosse et al., 2007), the *Repetition of Non-words Series* (Marinelli, 2010), and at the *Blending test* (Di Filippo et al., 2005) in the pseudoword condition (only a trend was present for the word condition). No difference was present at the *Digit Span* test (Wechsler, 2006). At any rate, it may be noted that, in most cases, the impairment was mild and (with the exception of the pseudoword condition of the *Blending test*) very few children showed frankly impaired performance in these tests.

**Table 2:** Performance of children with dyslexia and control readers in the *Test for the Diagnosis of Developmental Dysgraphia* (Angelelli et al., 2008).

	Control readers		Children with dyslexia				
	*M*	*SD*	*M*	*SD*	% path. perf.	*t*_(40)_	*p*
Regular words	-1.38	2.01	-2.93	3.85	41.18	1.68	n.s.
Regular non 1:1 words	0.05	0.60	-1.33	1.80	29.41	3.49	< 0.001
Ambiguous words	0.76	0.96	-1.04	1.36	23.53	4.98	< 0.001
Pseudo-words	-0.20	0.56	-1.73	2.55	35.29	2.86	< 0.01

Total	0.20	0.72	-2.04	1.80	47.06	5.50	< 0.001

Values indicate *z* scores as compared to normative values (negative values indicate lower performance). Path. perf., pathological performance. Regular words (*N* = 70) were with complete one-sound-to-one-letter correspondence; Non 1:1 regular words (*N* = 10) were words containing sounds that require syllabic conversion rules {i.e., the orthographic transcription of a consonant is determined by the vowel that follows it; for example [k] followed by /a/o/u/ = CA, CO, CU [e.g., CASA ( =home) and CUBO ( =cube)]; while [k] followed by /i/e/ = CHI, CHE [e.g., CHIESA ( =church)]}; ambiguous words (*N* = 55) were words with unpredictable transcription along the phonological-to-orthographic conversion route (e.g., [kwo] in [kwota], the quota: QUOTA but not *CUOTA); pseudo-words (*N* = 25) were stimuli without lexical status but with one-sound-to-one-letter correspondence.

**Table 3:** Performance of children with dyslexia and normal readers on visual attention and phonological/metaphonological tests.

Time	Control readers		Children with dyslexia				
	*M*	*SD*	*M*	*SD*	% path. perf.	*t*_(40)_	*p*
Visual attention span	0.44	0.70	-0.14	0.43	0.00	3.01	< 0.01
Phonological span	0.61	1.21	0.06	0.66	1.65	1.65	n.s.
Repetition of pseudo-word series	0.87	1.15	0.17	0.85	2.12	2.12	< 0.05
Blending test: words	0.73	0.94	0.05	1.31	1.95	1.95	0.06
Blending test: pseudo-words	0.64	1.10	-0.53	1.21	23.52	3.21	< 0.01

*For all tests, values indicate *z* scores based on normative values (negative values indicate lower performance). Path. perf., pathological performance.*

*The *Visual Attention Span* (Bosse et al., 2007) is a task in which children see on the PC screen for 200 ms an unpronounceable string of five consonants (e.g., R H S D M) that, as such, cannot be recoded phonologically and must report as many letters as possible. Each letter is presented 10 times appearing twice in each of the five positions. The task includes twenty items and was implemented by the E-prime 2 software. Normative data on Italian children are presented in Marinelli (2010).*

*Phonological span was assessed with the *Digit span task* of the WISC III (Wechsler, 2006).*

*In the *Repetition of Non-word Series,* ten lists of three bi-syllabic, 5-letter pseudo-words are read aloud by the examiner at a pace of about one every 2 s. The child is asked to repeat each list as accurately as possible immediately after presentation. Each correct non-word was awarded a point out for a maximum score of 30. Normative data on Italian children are presented in Marinelli (2010).*

*The *Blending Test* (Di Filippo et al., 2005) is a measure of phonological awareness. Words (or pseudo-words) are presented phoneme-by-phoneme through an audiotape at a rate of one per second. At the end of the sequence, the child has to repeat aloud the whole stimulus. Nineteen (five- to six-letter) words/pseudo-words are presented. For each stimulus, the correctly blended pairs of phonemes are counted, irrespective of whether repetition of the entire target is achieved. The maximum score is 83.*

#### Materials

Ninety-six 4-, 5-, and 6-letter words were selected from the *EPOS 2* database (Baldi and Traficante, 2005), based on the ease of recognition (words recognized by more than 90% of subjects) and high familiarity (familiarity estimated higher than 6 on a 7-point scale). Half of words were of high-frequency (mean = 215.4; *SD* = 142.2; range = 70–794) and half of low frequency (mean = 14.5; *SD* = 5.6; range = 7–28), according to the children words frequency corpus (Marconi et al., 1993). Both high- and low-frequency word subsets were made of 16 stimuli for each length (4-, 5-, and 6- letters). Subsets were matched for bigram frequency, contextual rules, presence of orthographic complexity (double consonants and cluster of consonants), familiarity (based on *EPOS 2*, Baldi and Traficante, 2005) and percentage of recognition (Baldi and Traficante, 2005). Words were also matched for neighborhood-size (Baldi and Traficante, 2005), but only within the subsets with the same number of letters (due to the high covariance between length and neighborhood-size characteristic of Italian).

For each subset, pronounceable strings such as DASU (16 stimuli for each length for a total of 48 items) were generated from half of the words, and unpronounceable stimuli such as RNGM (16 stimuli for each length for a total of 48 items) from the other half. Although unpronounceable, the letter stimuli were made only with bigrams really existing in the Italian orthography. Usually, studies compare a pronounceable non-word such as STRENG with a consonant string such as STPFM. However, in this example, not only is the first type of stimulus pronounceable and more orthographically similar to a real word than the second one, but it is also orthographically and phonologically regular, whereas the second is not. In the present study, we used only orthographically and phonologically regular bigrams in order to control for this aspect, at least at the bigram levels. For example, bigrams in VRSN are, respectively, in the words *aVRemo* (“we will have”), *oRSo* (“bear”), *SNello* (“slim”). Digrams SC, GL, GN, CH, which correspond to a single sound, were avoided.

Pronounceable strings were obtained changing vowels with other vowels, while unpronounceable strings were obtained changing vowels with consonants. Overall, there was the same number of “yes” and “no” responses; i.e., 96 real words (“yes” responses) matched with 96 non-words (“no” responses: 48 pronounceable and 48 unpronounceable). Items were randomized and presented in four blocks of 48 stimuli. Words and respective derived pseudowords or non-words did not appear in the same block. The order of presentation of the stimuli was randomized for each subject.

#### Procedure

Tests were carried out individually in a quiet room at the school of the children. Children performed a LDT, in which they had to decide whether or not a string of letters formed a legal Italian word.

Stimuli were printed in upper-case Courier new font, size 18, with a white color on a gray screen. Each item was preceded by a fixation point, which disappeared after 500 ms. After the appearance of the stimulus (that remained on the screen until the subject responded), there was a 1000 ms inter-trial interval. When the letter string appeared at the center of the PC screen, children had to push the right button on the keyboard as quickly and as accurately as possible if the stimulus was a word and the left one if it was not a word. The other buttons of the keyboard were hidden by means of a cardboard. A brief practice with 12 stimuli preceded the experiment. No feedback was provided. Children were allowed brief pauses between blocks.

Stimulus presentation and data recording were controlled with the E-Prime 2 software. The program recorded RTs and errors.

#### Data analysis

Invalid trials (due to technical problems), RTs below 250 ms and outliers (i.e., RTs exceeding the individual mean plus or minus 3 *SD*s) were excluded from the analyses. The percentage of excluded RTs was very small (for control readers: 0.47, 2.18, and 1.06% for unpronounceable non-words, pronounceable pseudowords and words, respectively; for children with dyslexia: 0.29, 1.82, and 1.06%, respectively). The RTs corresponding to errors were excluded from the analyses.

RTs were examined in order to check for the presence of the global factor in the data. In particular, the RAM (Faust et al., 1999) makes a number of testable predictions to detect the presence of global factor(s) in the data (see Results). When two groups vary for some general processing speed factor, larger group differences are expected in more difficult conditions (and smaller ones in an easier condition) over and above the specific effect of a given experimental manipulation; this is referred to as over-additivity effect (Faust et al., 1999). Over-additivity may modulate the group by condition interactions when two groups differ in general ability (Faust et al., 1999), as is the case for dyslexic and control readers. According to Faust et al. (1999), this effect can be controlled for by using various data transformations, including a *z* score transformation. For each participant, *z* scores are obtained by taking the RTs in each condition, subtracting their overall mean, and dividing them by the standard deviation across conditions (therefore, each individual has an average of 0 across conditions and a *SD* = 1). This transformation rescales individual performance to a common reference; hence, it allows controlling for global differences in information processing (Faust et al., 1999) while preserving the information regarding individual variability across experimental trials and conditions. Note that this transformation is appropriate only to open-scale measures, such as time, but not closed-scale measures such as accuracy. Interactions that are significant in both the raw score and *z*-transformed score analyses indicate the selective influence of a given parameter; in contrast, interactions that are significant only in the raw data analyses, but not on those with the *z*-transformed values, indicate the presence of spurious interactions (due to over-additivity effects; Faust et al., 1999).

Three separate analysis of variances (ANOVAs) were carried out to examine the effect of pronounceability (non-words vs. pseudowords), frequency (high- vs. low-frequency words) and lexicality (pseudowords vs. words), respectively. In each of these analyses, group (dyslexic vs. control children) was entered as between-subject factor and length (4-, 5-, and 6-letter stimuli) as repeated measure. Separate ANOVAs were carried out on percentages of errors, RT raw data (r) and RT *z*-transformed data (z). For the sake of presentation, the two latter types of analyses will be presented together (using raw RT means to illustrate effects); this will allow highlighting which group by condition interactions are genuine and which can be ascribed to the over-additivity effect. Whenever appropriate, means were compared with the *a posteriori* Tukey HSD test.

### RESULTS

#### Analysis of global factor(s)

The RAM (Faust et al., 1999) predicts a linear relationship between: (i) the condition means of two groups of children (e.g., dyslexic and control readers) who vary in overall information processing rate; (ii) the condition means of the overall group and the standard deviation in the same conditions (i.e., that more difficult conditions will generate greater variability).

**FIGURE 1:**
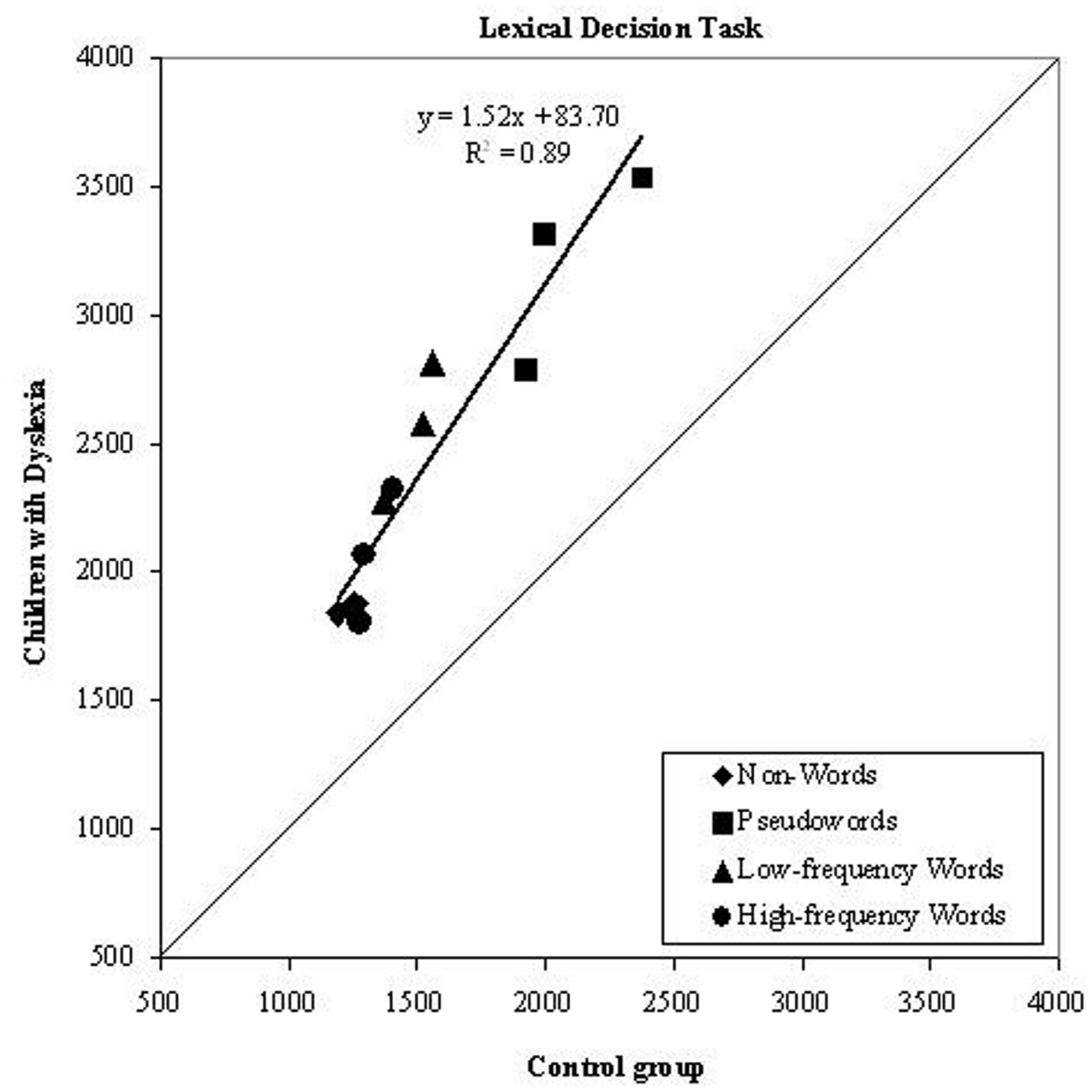
**Experiment 1.** Dyslexics’ condition means in the lexical decision task are plotted as a function of the control readers’ means (symbols as described in the figure; the three symbols per condition represent word lengths). The diagonal line (slope = 1) represents equal RTs for dyslexic and control readers. Note that all data points lie above the diagonal line indicating that children with dyslexia were slower than controls in all conditions. All data points are well fit by a single regression line.

As it can be seen in **Figure 1**, condition means for the dyslexic group were linearly related to those of control readers. This pattern indicates that a global factor (which explains a large proportion of variance, i.e., *r^2^* = 0.89) accounts well for the slowness of children with dyslexia across all experimental conditions; namely, condition means for high- and low-frequency words, pronounceable pseudowords and unpronounceable non-words were all well fit by the same regression line. The slope is 1.52, indicating that children with dyslexia were 52% slower than control readers in performing the task.

**FIGURE 2:**
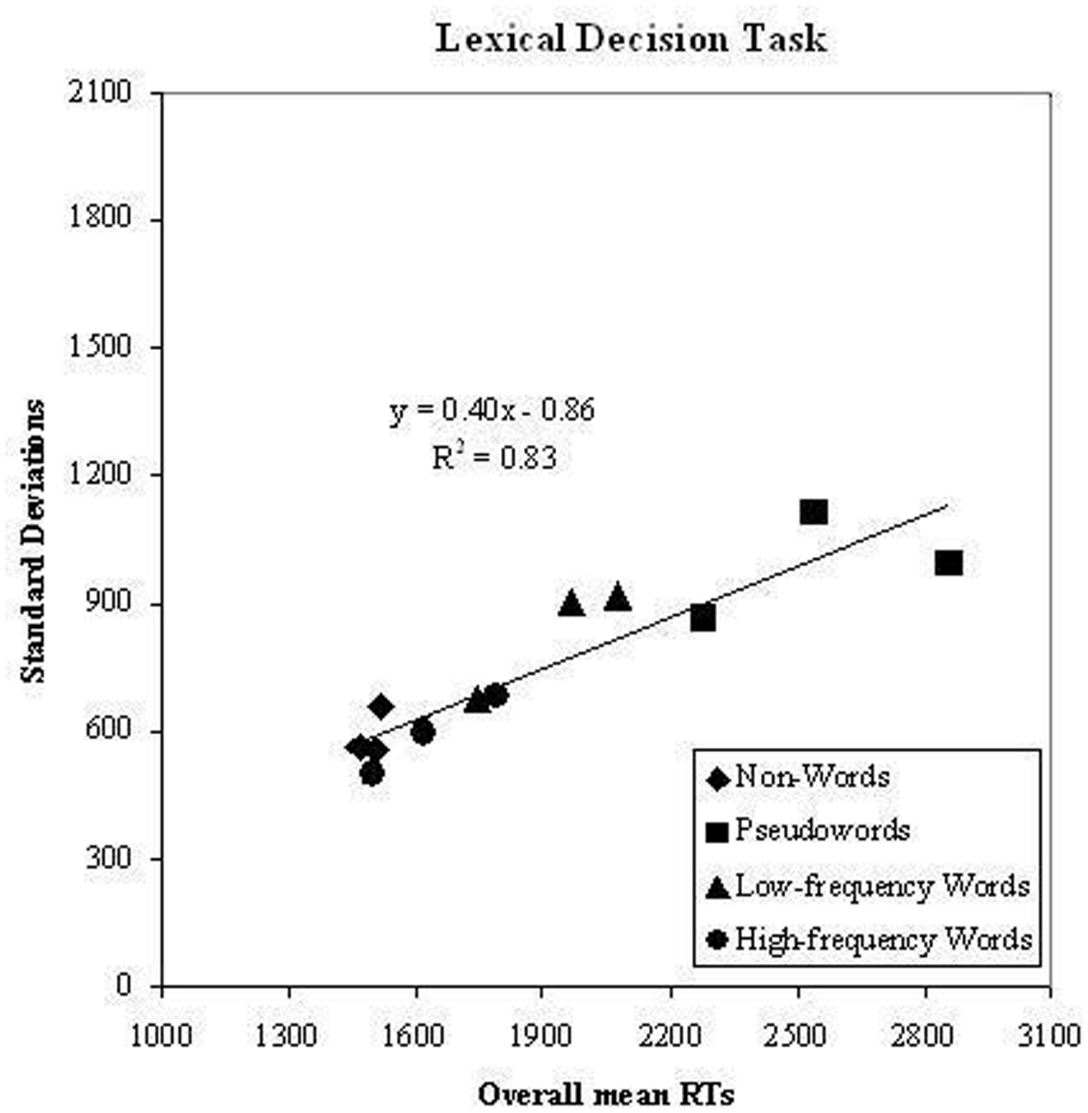
**Experiment 1.** Condition means in the lexical decision task and standard deviation on the corresponding conditions are plotted against each other; data refer to the whole group of participants (dyslexic and control readers). Symbols are described in the figure; the three symbols per condition represent word lengths.

The test of the second prediction is presented in **Figure 2**; the means of the overall sample of children (dyslexic and control readers) for all experimental conditions are plotted against the respective standard deviation in the same conditions. A linear relationship between means and standard deviation (with a 0.40 slope) was present accounting for a substantial amount of variance (*r^2^*= 0.83).

Due to the presence of a global factor in the data, in accordance with the RAM, the ANOVAs were performed also on *z*-transformed RTs in order to determine whether stimulus pronounceability, as well as frequency and lexicality, have a specific role in modulating group differences over and above the variance accounted for by the global factor.

#### Analysis of variance

**FIGURE 3:**
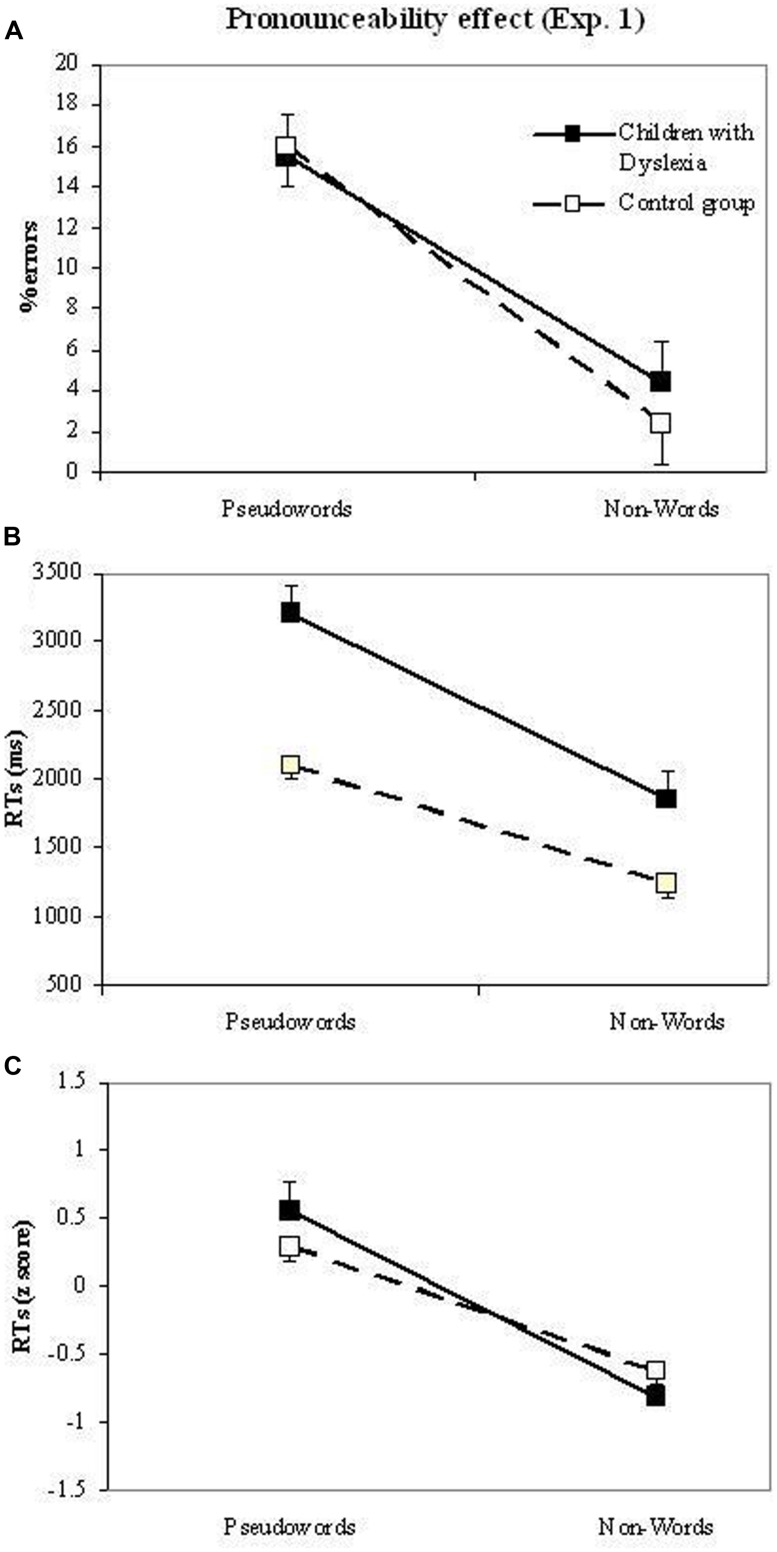
**Experiment 1.** Performance on pseudowords and non-words (pronounceability effect) of dyslexic and control readers in the lexical decision task. The three plots report data in terms of errors (plot a), RT raw data (plot b) and *z*-transformed RT data (plot c).

***Pronounceability.* Figure 3** shows the relevant means of the pronounceability effect in terms of errors, raw RTs and *z*-transformed RTs, separately for dyslexic and control readers.

The ANOVA on errors showed the main effect of pronounceability (*F*_(1,39)_ = 45.9, *p* < 0.001), with higher percentages of errors for pseudowords (15.8%) than non-words (3.4%). The length and group main effects as well as the interactions between these variables were all not significant (all *F*s about 1).

The ANOVAs on RTs showed the significance of the main effects of group (*F_r_*_(1,39)_ = 28.0, *p* < 0.001; the group effect is by definition nil in the analyses on *z*-transformed data), pronounceability (*F_r_*_(1,39)_ = 100.1, *p* < 0.001; *F_z_*_(1,39)_ = 45.0, *p* < 0.001), and length (*F_r_*_(2,78)_ = 13.9, *p* < 0.001; *F*_z(2,78)_ = 8.9, *p* < 0.001), with shorter RTs for control readers with respect to children with dyslexia (1671 vs. 2532 ms), for non-words compared to pseudowords (1546 vs. 2656 ms) and for shorter stimuli compared to longer stimuli (1938, 2101, and 2264 ms for 4-, 5-, and 6-letter stimuli, respectively). Pronounceability interacted with group in the raw data analysis (*F_r_*_(1,39)_ = 5.1, *p* < 0.05), but the effect was not significant in the analysis with *z*-transformed data (*F_z_*_(1,39)_ = 1.9, n.s.), indicating that the interaction with the raw data was due to the influence of over-additivity. All other interactions with the group factor were not significant. Pronounceability interacted with length (*F_r_*_(2,78)_ = 8.5, *p* < 0.001; *F_z_*_(2,78)_ = 7.3, *p* < 0.001): length effects were present for pseudowords (mean increase per letter = 301 ms), but not for non-words (mean increase per letter = 25 ms).

***Frequency.*** The ANOVA on errors showed the significance of the main effects of group (*F*_(1,39)_ = 23.8, *p* < 0.001) and frequency (*F*_(1,39)_ = 79.8, *p* < 0.001), with higher percentages of errors for children with dyslexia (15.7%) than control (5.8%) readers and for low- (16.1%) than for high-frequency (5.4%) words. Frequency interacted with length (*F*_(2,78)_ = 4.2, *p* < 0.05), with a larger frequency effect for shorter than longer words: the difference between high- and low-frequency words was 13.9, 10.5, and 8.2% for 4-, 5-, and 6- letter words, respectively. Frequency also interacted with group (*F*_(1,39)_ = 25.2, *p* < 0.001), with a larger frequency effect for dyslexic than control readers (difference between low- and high-frequency words = 16.7 and 4.7% in the two groups, respectively), and a significant group difference for low- (15.9%, *p* < 0.001) but not high-frequency words (3.9%, n.s.).

The ANOVAs on RTs showed the significance of the main effects of group (*F*_r(1,39)_ = 32.3, *p* < 0.001), frequency (*F_r_*_(1,39)_ = 65.9, *p* < 0.001; *F_z_*_(1,39)_ = 67.7, *p* < 0.0001) and length (*F_r_*_(2,78)_ = 20.6, *p* < 0.001; *F_z_*_(2,78)_ = 15.7, *p* < 0.001), with shorter RTs for control readers than for children with dyslexia (1406 vs. 2310 ms), for high- than low-frequency words (1695 vs. 2021 ms) and for shorter than longer words (1682, 1867, and 2024 ms for 4-, 5-, and 6-letter words, respectively). Group interacted with length and frequency in the raw data (respectively: *F_r_*_(2,78)_ = 5.9, *p* < 0.01; *F_r_*_(1,39)_ = 17.3, *p* < 0.001), but the interactions disappeared in the analysis on *z*-transformed data, indicating that they were due to over-additivity in the data. All other interactions with the group factor were not significant.

***Lexicality.*** The ANOVA on errors showed the significance of the lexicality factor (*F*_(1,39)_ = 5.2, *p* < 0.05), with higher percentages of errors for pseudowords (15.8%) than words (10.8%). The group main effect approached significance (*F*_(1,39)_ = 4.0, *p* = 0.053) with a tendency for children with dyslexia to make more errors (15.6%) than control readers (10.9%). The lexicality by group interaction was significant (*F*_(1,39)_ = 5.6, *p*< 0.05) indicating the presence of a lexicality effect in control readers (difference between words and pseudowords = 10.1%, *p* < 0.01), but not for children with dyslexia (difference between words and pseudowords = –0.2%, n.s.). Groups had a similar performance in the case of pseudowords (16.0% of errors for control readers and 15.5% for children with dyslexia), while, in the case of words, control children produced fewer errors than children with dyslexia (5.8 vs. 15.7% respectively; *p* < 0.05).

The ANOVAs on RTs showed the main effects of group (*F_r_*_(1,39)_ = 31.9, *p* < 0.001), lexicality (*F_r_*_(1,39)_ = 80.9, *p* < 0.001; *F_z_*_(1,39)_ = 1.79, n.s.) and length (*F_r_*_(2,78)_ = 23.6, *p* < 0.001; *F_z_*_(2,78)_ = 33.5, *p* < 0.001). RTs were shorter for control (1753 ms) than dyslexic (2761 ms) readers, for words (1858 ms) than for pseudowords (2656 ms), and for shorter than longer stimuli (2019, 2261, and 2491 ms for 4-, 5-, and 6-letter stimuli, respectively). Group interacted with length (*F*_(2,78)_ = 3.8, *p* < 0.05), but the interaction disappeared in the *z* score analysis (*F_z_*_(2,78)_ = 0.56, n.s.), in keeping with the idea that it was due to over-additivity in the data. Group did not interact with lexicality (*F* about 1) in both raw and *z*-transformed data analyses, with similar lexicality effects for dyslexic and control readers (mean effect = 693 and 902 ms in the two groups, respectively).

### DISCUSSION

Both groups of children were faster and more accurate at rejecting unpronounceable non-words than pronounceable pseudowords. This finding is consistent with previous studies (e.g., Holcomb and Neville, 1990; Forster et al., 2003; Ratcliff et al., 2004; Evans et al., 2012). As foils are more word-like (as in the case of pseudowords vs. non-words) the LDT proves more difficult. This might depend from several factors such as: (i) the increase of orthographic and phonological overlap between words and foils; (ii) foils producing more activation of similar words in the orthographic input lexicon; (iii) fewer sources of information being available to solve the task. In fact, for non-words, all procedures (semantic, lexical, and sub-lexical) are in favor of a “non-word” response (similarly to what happens for words for which all procedures are in favor of a “word” response). By contrast, in the case of pseudowords, the lexical and semantic routes favor a “no” response, while the sub-lexical procedure a “yes” response.

Pronounceability did not interact with group in the case of errors, indicating a similar pattern in the two groups. With regard to RTs, pronounceability interacted with group in the raw data, but not when over-additivity was taken into account in the *z*-transformed analysis: the larger effect of pronounceability among children with dyslexia was due to the presence of over-additivity in the data and the two groups showed a similar disadvantage in rejecting pseudowords compared to non-words.

Critically for the purpose of this study was to examine if performance on unpronounceable strings maps onto the global factor which accounts for the differences in performance between dyslexic and control readers. Consistent with the predictions of the RAM, condition means for the dyslexic group were linearly related to those of control readers. This pattern indicates that a single global factor accounts quite well for the slowness of children with dyslexia across all experimental conditions. Condition means for pronounceable pseudowords and unpronounceable non-words essentially showed the same result (i.e., they were all well fit by the same regression line). The children with dyslexia’s impairment was evident when they had to process strings not only of pronounceable stimuli (such as words and pseudowords) but also unpronounceable stimuli (i.e., when they had to decide that a string of consonants was not a word), a deficit well accounted for by a single global factor.

Consistently with previous studies of Italian children with dyslexia using reading (e.g., Barca et al., 2006) and LDT (e.g., Marinelli et al., 2011), results highlighted a frequency effect among both children with dyslexia and control readers. This pattern indicates that children with dyslexia benefit from lexical activation in performing the task also in a highly regular orthography, such as Italian. The size of the frequency effect was actually larger for dyslexic than control readers but this difference disappeared when over-additivity was taken into account. The effect of lexicality did not interact with group in RTs (in both raw and *z*-transformed data), but only in errors, due to the absence of group differences for pseudowords (but only for words). Also this finding confirms previous evidence on Italian children with dyslexia (Zoccolotti et al., 2008).

## EXPERIMENT 2

The first experiment supports the hypothesis that the difficulty in processing strings of letters accounts for a large amount of children with dyslexia’s impairment, irrespective of the pronounceability of the strings. The second experiment, based on the Reicher–Wheeler paradigm, tests the ability of children with dyslexia to discriminate a target letter from a competitor in the context of strings of letters similar to the stimuli used with the LDT, i.e., words, pseudowords, and non-words. This task allows detecting the sensitivity of the two groups of children to use the prime pronounceability and lexical information to favor graphemic processing while responding is limited to the forced-choice discrimination of a target letter. If the difficulty of children with dyslexia is specifically linked to the ongoing simultaneous processing of a letter string (as in the LTD), no deficit should be present in this condition. At the same time, the possibility to test the sensitivity to context provides a further test of the distinction between a pre-lexical graphemic level and orthographic–phonological binding interpretation. Based on the orthographic–phonological binding hypothesis, the PSE (and the WSE) is expected for control children but not for children with dyslexia. Based on the letter string graphemic hypothesis, no difference in these effects is expected between the two groups of children.

### METHOD

#### Participants

Same as Experiment 1.

#### Materials

Three groups of 4-letter stimuli were presented: 48 words (e.g., VISO, “face”), 48 pseudowords (e.g., VESI), and 48 letter strings (e.g., VRSN). Each derived pseudoword or non-word maintained two of letters from the original word.

All words had a CVCV structure and were selected from the Elementary lexicon by Marconi et al. (1993) and were high-frequency words (*M* = 181/1 million, *SD* = 261), with high rate of familiarity (Baldi and Traficante, 2005; *M* = 6.8/7 rating scale points, *SD* = 0.08), easy to recognize as real Italian words (wordlikeness; Baldi and Traficante, 2005; *M* = 99.35% of correct lexical judgment by adult proficient readers, *SD* = 0.7), and with a mean of four orthographic neighbors (Baldi and Traficante, 2005; *M* = 3.9, *SD* = 1.7).

Pseudowords were made from words, by changing the two vowels of the base stimulus. As mentioned above, letter strings were made of legal digrams, i.e., sequences of two letters that can be found in real Italian words. Target letters in first and third position were minimal phonological pairs (i.e., phoneme that differs for only one phonological feature, such as P–B, L–R, N–M) in order to emphasize the role of phonological decoding. The competitor letter was never in the multi-letter string and, in the case of substitution in the string, the competitor did not produce a lexical orthographic neighbor of target itself. The number of visually similar competitors (53%; e.g., P–B, N–M) was matched in each position. Target letters in second and fourth positions were vowels; so, in this case, it was not possible to use minimal pairs. Moreover, due to the ortho-phonotactic structure of Italian language, in which ending is always a vowel (a, e, i, o), stimuli with targets in fourth position were presented, but considered as fillers, because, in the case of words, they often produced other words, differently from the condition of consonant targets. However, they were presented to avoid children to focus their attention only on the first three letter positions.

For each stimulus type there were 16 targets in first, 16 in second, and 16 in third position, respectively, for a total of 48 stimuli per group and a grand-total of 144 stimuli. Filler stimuli with target in fourth position were eight in each group for a total of 24 stimuli. The overall number of stimuli was 168. Three blocks of stimuli were made, separated by a brief pause, in order to avoid attention decrease. Three blocks were matched for word frequency, familiarity, wordlikeness, and number of orthographic neighbors. In each block there was the same number of words, pseudowords, and non-words, equally distributed for each target letter position, avoiding that base-words and derived pseudowords and non-words were presented in the same block.

#### Procedure

Children made the task in a quiet room, sitting at about 54 cm from the screen. Stimuli were presented in Courier New, size 18 pt, in upper-case, in white foreground on gray background.

**FIGURE 4:**
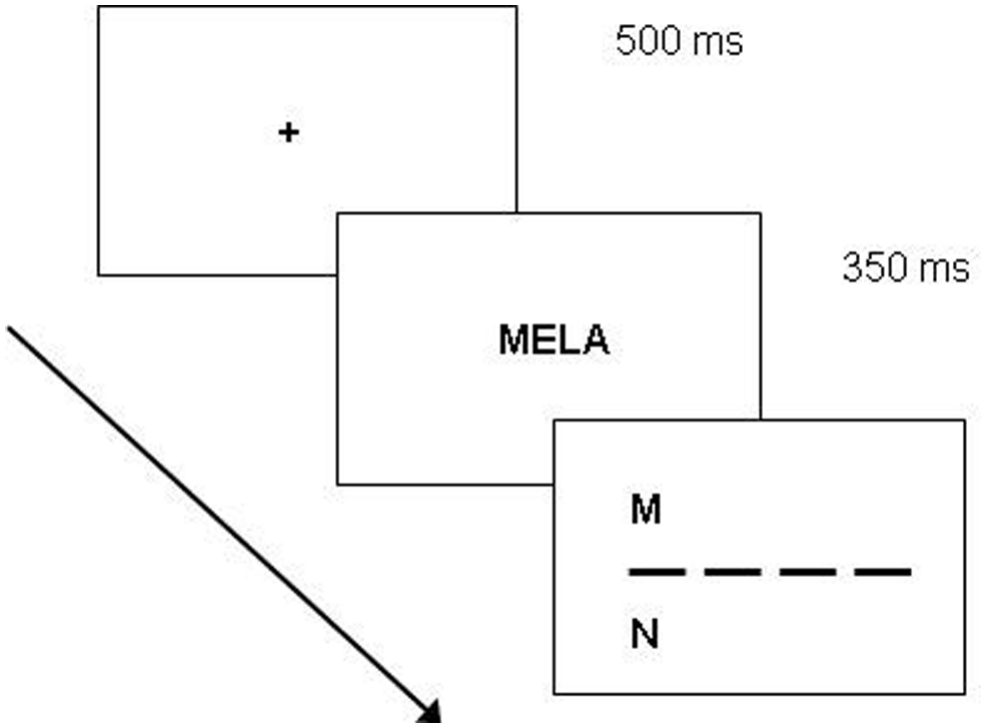
**Experiments 2.** Time-course of the trial in the Reicher–Wheeler paradigm.

The trial sequence started with a get-ready display (500 ms), followed by the presentation of the multi-letter string (either word, pseudoword, or non-word) for 350 ms^1^ and by the target letter display, which lasted until the forced-choice discrimination between target and competitor was made (**Figure 4**). The response was given by pressing one of two buttons of the keyboard: the “Up” button to choose the letter in the upper part of the display, the “Down” button for the letter in the bottom part. Correct responses were in half of the cases the “Up” choice.

Ten training stimuli were presented at the beginning of the experimental session. Three blocks of stimuli followed in a fully randomized order between blocks and within each block.

The program automatically recoded the responses of the participant; percentages of errors and RTs (only to correct responses) were used as dependent measures. Outliers (i.e., RTs 3 *SD*s below the mean) and invalid responses (i.e., responses faster than 250 ms or RTs not recorded correctly for technical problem) were excluded from the analysis.

### RESULTS

Invalid responses and outliers were about 2.52% in children with dyslexia and 2.04% for typically developing children.

#### Analysis of global factor(s)

**FIGURE 5:**
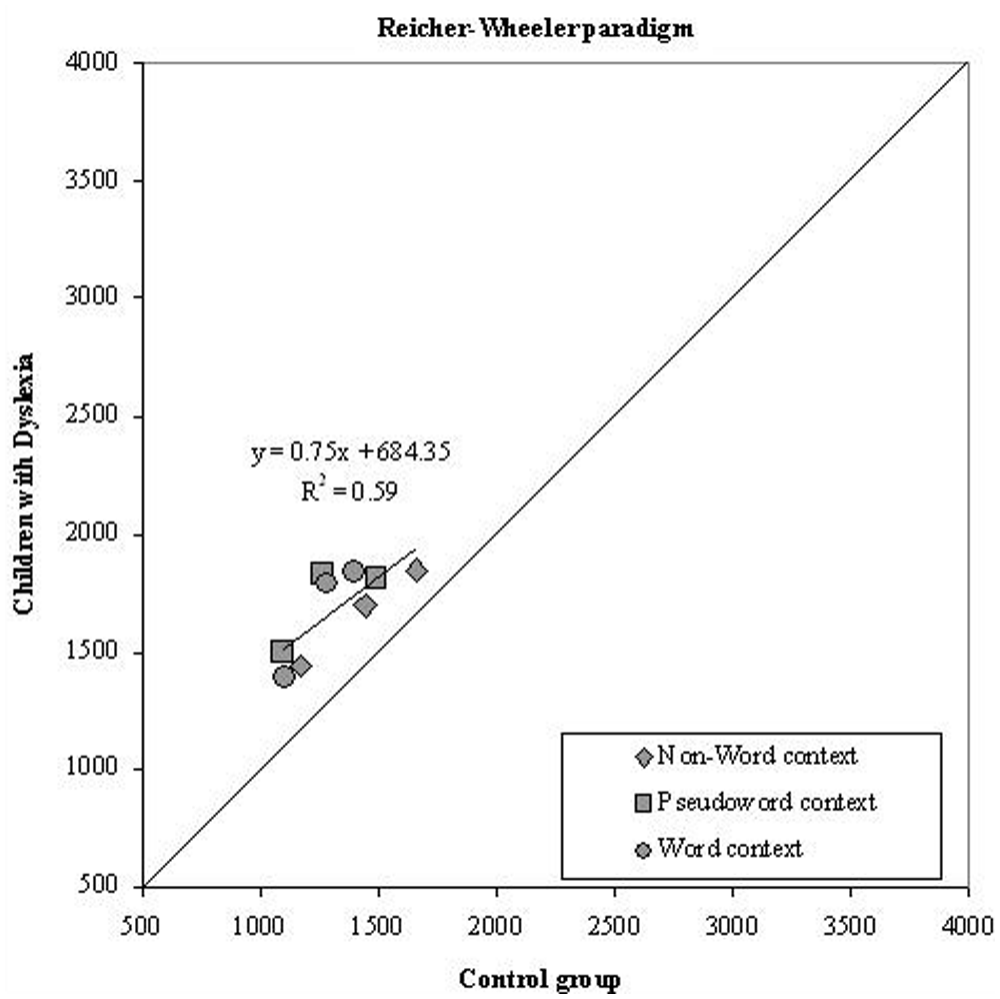
**Experiment 2.** Dyslexics’ condition means in the Reicher–Wheeler paradigm are plotted as a function of the control readers’ means (symbols as described in the figure; the three symbols per condition represent letter positions). The diagonal line (slope = 1) represents equal RTs for dyslexic and control readers.

Before proceeding to the analysis of specific effects, we examined data for the possible presence of global components in the differences between the two groups of children (Faust et al., 1999). We first tested the prediction of a linear relationship between the means of the two groups for conditions that varied in overall information processing rate. Dyslexics’ and skilled readers’ condition means are plotted against each other in **Figure 5**, separately for each experimental condition in the Reicher–Wheeler paradigm.

Note that all data points are above the diagonal line (which indicates the benchmark for identical performance of the two groups); thus, children with dyslexia tended to be slower than typically developing readers across all conditions. In the Reicher–Wheeler paradigm, the percentage of variance accounted for by the regression line was moderate (59%) and the slope was less than unity (*b* = 0.75) indicating no over-additivity effect. Thus, in this case, the group differences appear entirely due to the intercept value (i.e., to a constant value).

**FIGURE 6:**
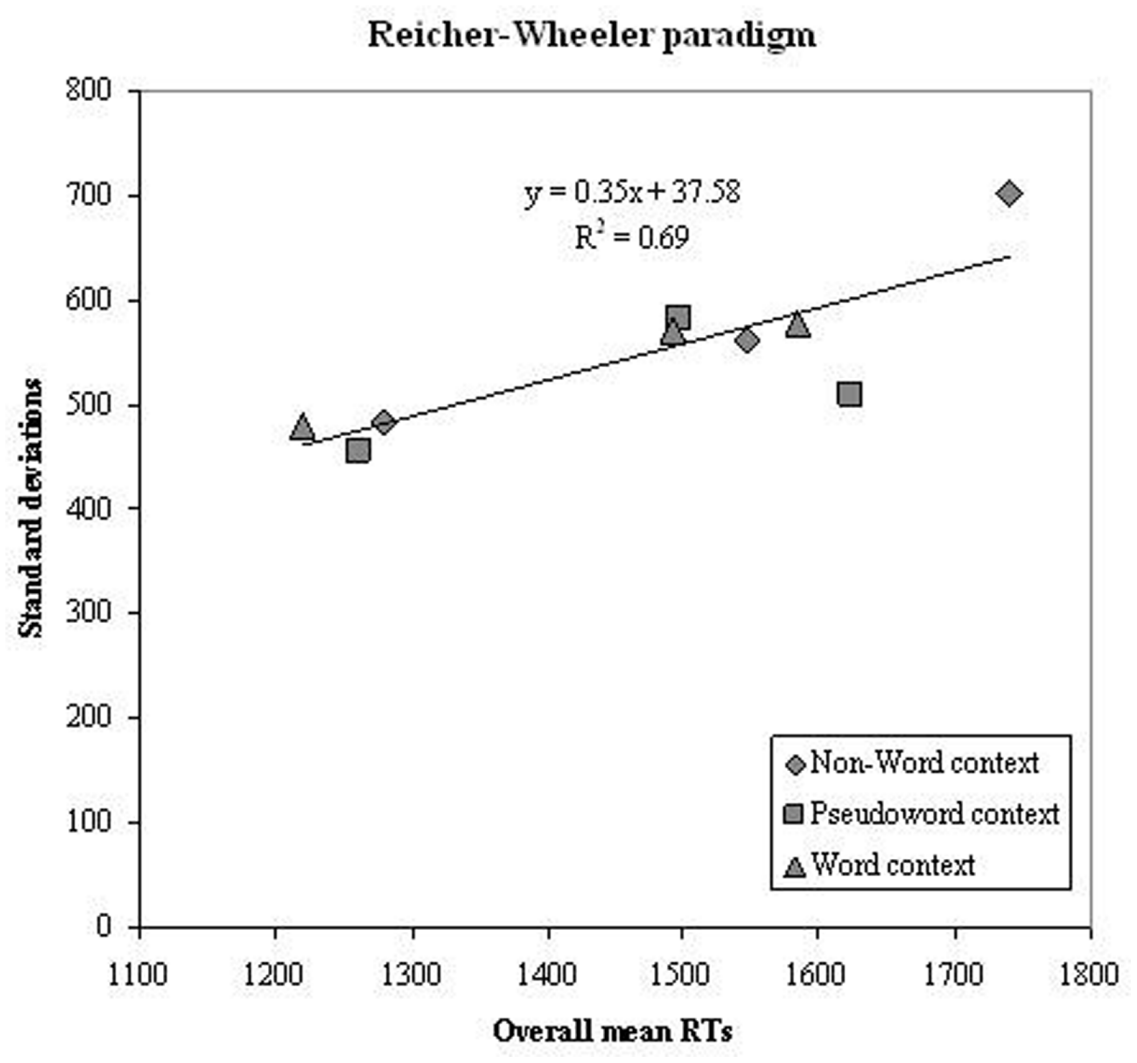
**Experiment 2.** Condition means in the Reicher–Wheeler paradigm and standard deviation on the corresponding conditions are plotted against each other; data refer to the whole group of participants (dyslexic and control readers). Symbols are described in the figure; the three symbols per condition represent letter positions.

Successively, we tested the prediction of a linear relationship between overall group means and standard deviation in the same conditions for the group as a whole. **Figure 6** reports the mean of the overall sample against the standard deviation for each corresponding experimental condition. The regression line was not very steep (0.35) and the percentage of variance explained for the conditions of the Reicher–Wheeler paradigm was moderate (69%).

As a global factor was not detected for the conditions of the Reicher–Wheeler paradigm, the *z* score transformation was not used and only standard RT analyses were carried out.

A mixed ANOVA with group (children with dyslexia vs. typically developing children) as a between-subject factor and context (words, pseudowords, and non-words) and position (first, second, and third position) as repeated measures was carried out on the percentages of errors in letter recognition. Significant interactions were explored with the *a posteriori* Tukey HSD.

The ANOVA showed the main effects of group (*F*_(1,39)_ = 5.71, *p* < 0.05), context (*F*_(2,78)_ = 122.46, *p* < 0.001), and position (*F*_(2,78)_ = 26.75, *p* < 0.001), as children with dyslexia made more letter recognition errors (17.9%) than typically developing children (10.6%), letters were recognized less well in the context of non-words (23.1%) than in the pseudoword (11.1%) and word (8.5%) contexts, and errors increased from first (9.5%) to second (15.3%) and third (18%) position.

All two-way interactions were significant. The group by context (*F*_(2,78)_ = 16.26, *p* < 0.001) indicated the presence of both the PSE (non-word context: 29.7%; pseudoword context: 14.3%; Tukey test: *p* < 0.001) and the WSE (word context: 9.5%; *p* = 0.02) in children with dyslexia. In typically developing children only the PSE reached the significance level (non-word context: 16.5%; pseudoword context: 7.8%; *p* < 0.001), while letter recognition errors in the word context (7.6%) did not differ from those in the pseudoword context. As for the group by position interaction (*F*_(2,78)_ = 5.40, *p* < 0.01), in children with dyslexia there was an increasing amount of letter recognition errors from the first (11.2%) to the second (18.9%) position (*p* < 0.001) and from the first to the third (23.5%) position (*p* < 0.001). For typically developing children the only significant difference was between the first (7.9%) and third position (12.4%; *p* = 0.04), while the second position (11.7%) did not differ from the others. The context by position interaction (*F*_(4,156)_ = 9.36, *p* < 0.001) indicated that in the non-word context there was an increasing amount of errors from the first (13.6%) to the second (25.2%) position (*p* < 0.01) and from the first to the third position (30.6%, *p* < 0.01), while the difference between the second and the third position did not reach significance level. In the pseudoword context, the first position was associated to a lower amount of errors (6.7%) than the third position (14%, *p* = 0.02), while percentage of errors in second position (12.5%) did not differ from the others. In the word context, letter recognition errors were low and similar in every position: 8.2% in first, 8.1% in second, and 9.3% in third position, respectively.

The ANOVA on RTs showed the main effects of group (*F*_(1,39)_ = 6.01, *p* = 0.019), and position (*F*_(2,78)_ = 42.33, *p* < 0.0001), with longer RTs for children with dyslexia (1681 ms) than typically developing children (1323 ms), and for letters in third (1676 ms) compared to second (1550 ms, *p* < 0.001) and first (1281 ms, *p* < 0.01) positions, but no main effect of context (*F*_(2,78)_ = 2.19, *p* = 0.119). Group interacted with context (*F*_(2,78)_ = 4.24, *p* < 0.05): there were smaller group differences in the non-word context (difference = 235 ms) compared to the pseudoword (difference = 427 ms) and word (difference = 411 ms) contexts. However, none of these differences reached significance at the *post hoc* analyses. In typically developing readers there was a detectable PSE (difference between the non-word and pseudoword contexts = 147 ms; *p* = 0.05), but no WSE (difference between pseudoword and word context = 15 ms). For children with dyslexia neither the PSE (difference between non-word and pseudoword contexts = –51 ms) nor the WSE (difference between pseudoword and word context = 39 ms) were present.

### DISCUSSION

In the case of accuracy data, the results indicated a robust PSE in both groups of children, while the WSE was present only among children with dyslexia. Thus, accuracy in letter discrimination in young Italian readers, and remarkably also in children with dyslexia, was influenced by the ortho-phono-tactic regularity of the letter string. The results were generally less clear-cut in the case of RTs where the main effect of context was not significant. However, a significant PSE was detected in the case of typically developing children.

The present pattern of findings shares a number of similarities to the previous results on French children reported by Grainger et al. (2003). They found a large PSE effect in both dyslexic and reading-matched control children but no WSE for either group of children (while the WSE was present with the same type of stimulus materials in a group of adult readers). They proposed that the joint presence of PSE and absence of WSE favors a sublexical–orthographic interpretation, based on the greater familiarity of letter combinations in pseudowords compared to non-words. Pseudowords provide letter clusters which represent typical orthographic contexts for a given letter in a given position. Within this interpretation, children with dyslexia show a spared ability to use such sublexical–orthographic information to shape their performance in letter recognition. This pattern is at odds with the orthographic–phonological binding interpretation while it is consistent with a pre-lexical graphemic interpretation.

It is worth noting that in our study the facilitating role of lexical activation producing the WSE emerged just in children with dyslexia. This is in keeping with studies that found also in Italian, a language with a very consistent orthography, evidence of the activation of lexical representations in young readers. Several Italian studies (Barca et al., 2006; Paizi et al., 2013) showed lexical involvement in reading of children with and without dyslexia. The authors suggested that children might rely more on the lexical route when the non-lexical route is not automatized yet. Results observed in children with dyslexia through the Reicher–Wheeler paradigm, in the present study, seem consistent with this hypothesis.

A general question addressed by Experiment 2 was whether performance in the Reicher–Wheeler paradigm would generate global group differences as reported for LDTs. The LDT used in Experiment 1 clearly yielded global differences in performance as previously reported with similar materials (e.g., Di Filippo et al., 2006; Paizi et al., 2013). In the case of the Reicher–Wheeler paradigm group differences were present although generally much smaller than those observed in the case of the LDT. Critically, when the RAM was applied to the time measures, group differences in RTs did not grow as a function of condition difficulty as expected in the case of a global factor (and an over-additivity effect). Indeed, the slope of the linear regression was smaller than unity. Thus, the small group differences were expressed by a constant value (intercept). How can this effect be explained? De Luca et al. (2010) noted that children with dyslexia have less practice with orthographic materials and proposed that this factor may be sufficient to explain the small deficit in letter-bigram tasks. The role of familiarity has been systematically tested by Valdois et al. (2012) who examined the performance on letter string, digit string, and color string processing; dyslexic children were impaired in the first two tasks but performed as controls in the color report task. This pattern is consistent with a familiarity account while is inconsistent with a visual-to-phonological-code interpretation. Overall, this pattern of findings is in keeping with the idea that a selective deficit in children with dyslexia is present only when the task requires the entire string of orthographic stimuli to be simultaneously processed. By contrast, it has repeatedly been shown that children with dyslexia are not (or minimally) impaired in the processing of single letters or bigrams (e.g., Bosse et al., 2007; Martelli et al., 2009; De Luca et al., 2010) or when the set of target letters is presented sequentially (Lassus-Sangosse et al., 2008).

## GENERAL DISCUSSION

The results from the LDT in Experiment 1 indicated that a single global factor explained the performance with orthographic strings, independent from stimulus pronounceability, as well as frequency and lexicality. The children with dyslexia’s impairment was evident (and of a comparable size) when they had to process strings, not only of pronounceable stimuli (such as words and pseudowords) as already reported in previous studies (e.g., Di Filippo et al., 2006; Marinelli et al., 2011; Paizi et al., 2013), but also of unpronounceable stimuli, a deficit well accounted for by the same global factor. Thus, the present study adds a new piece of information to the understanding of the nature of the global component affecting performance of children with dyslexia. Previous studies indicated that a single global factor explains the deficit of children with dyslexia in making lexical decisions and reading words and pseudowords, i.e., independent of word frequency and lexicality (Di Filippo et al., 2006; Zoccolotti et al., 2008; Marinelli et al., 2011), but not in dealing with pictorial stimuli (Di Filippo et al., 2006; Zoccolotti et al., 2008) or stimuli in the auditory modality (Marinelli et al., 2011). The present study adds to this picture that the global factor is independent not only from the lexical status of the stimulus, but also from the pronounceability of the letter string: when the over-additivity effect was controlled for, the deficit of children with dyslexia in the LDT was detectable, and of a comparable size, when rejecting pronounceable pseudowords or unpronounceable non-words. Therefore, the present findings are consistent with the proposal that an impairment in pre-lexical graphemic analysis (i.e., in forming a graphemic description of the letter string) is a core deficit in developmental dyslexia (Zoccolotti et al., 2008); by contrast, they do not support the idea that the deficit in dyslexia is due to an inability to bind orthographic and phonological information (Ziegler et al., 2010; van den Broeck and Geudens, 2012), not even in Italian, a language with very consistent orthography.

The RT data from the Reicher–Wheeler paradigm indicated that group differences in this task did not generate global differences between children with dyslexia and control readers. These data are generally in keeping with previous observations by De Luca et al. (2010) indicating that children with dyslexia were only mildly affected in tasks requiring the naming or matching of individual letters, bigrams or two-letter syllables and no over-additivity effect was present for these tasks. Therefore, it appears that the global factor accounting for the impairment of children with dyslexia is present when the child processes a (relatively long) string of letters in parallel, not when the task concerns isolated letters. The present results add to this picture that, even if the processing of a letter string is slowed down in these children, they can take advantage from the ortho-phono-tactic information deriving from such processing in discriminating a subsequent isolated target letter from a competitor; i.e., they showed a clear PSE (at least in the case of accuracy) in the Reicher–Wheeler paradigm. This differentiation can be appreciated most clearly by comparing the performance in making a lexical decision on pseudowords with that of recognizing a target letter in the presence of a pseudoword context. In the first condition, children with dyslexia were severely impaired in both accuracy and speed; in the second, they were more accurate than in the case of a four-letter non-word context (i.e., they had a PSE) and the group difference with typically developing children was quantitatively quite small. Therefore, when cognitive tasks (e.g., lexical decision, naming, semantic categorization, etc.) are to be applied to letter strings as a whole, children with dyslexia are in difficulty. On the contrary, when tasks involve isolated letter processing, also these children can make use of the ortho-phono-tactic information derived from a previously seen letter string. This spared ability appears inconsistent with the idea that children with dyslexia suffer from a selective deficit in orthographic–phonological binding. By contrast, it is consistent with a pre-lexical graphemic interpretation; according to this view, online simultaneous processing of multi-letter elements is generally impaired. However, if sufficient time is given for processing a letter string, children with dyslexia may effectively use its ortho-phono-tactic information to modulate orthographic processing of isolated letters.

The present findings are in keeping with the available information on the characteristics of the VWFA. Thus, neuroimaging studies indicate that the VWFA is activated not only by orthographically legal stimuli, such as words and pronounceable pseudowords, but also by illegal letter strings (e.g., Cohen et al., 2002). There is clear evidence that event-related potentials (ERPs) recorded at posterior sites within the 150–250 ms time window at fronto-central, central, and temporo-parietal sites (typically in the form of the N200) are modulated by orthographic information. This finding is consistent with the hypothesis that the word form system analyzes visual linguistic stimuli at a pre-lexical level while information concerning lexical status and meaning is processed through additional neural systems (Bentin et al., 1999). In ERP studies, the VWFA does not generally differentiate between pseudowords and words (Hagoort et al., 1999) and no difference in N200 amplitude for these two stimuli is found (e.g., Tagamets et al., 2000 for a fMRI study). With regard to non-words, in some reports, the N200 was larger for non-words than for words (Compton et al., 1991; McCandliss et al., 1997), whereas, in others, the opposite pattern was reported (Cohen et al., 2000; Dehaene et al., 2002; Grossi and Coch, 2005) or no difference between legal and illegal orthographic letter strings was detected (e.g., Bentin et al., 1999). Some inconsistencies between studies may depend from differences in the experimental task. In fact, studies used several experimental paradigms: a letter search task (Ziegler et al., 1997), a letter-in-string identification task (Coch and Mitra, 2010), a LDT (Rosazza et al., 2009; Massol et al., 2011), and a rhyme judgment task (Bentin et al., 1999). In general, research comparing the processing of consonant strings and pronounceable pseudowords reports a divergence in the ERP waveforms as a function of target type only starting at around 200–250 ms post-target onset (Ziegler et al., 1997; Rosazza et al., 2009; Massol et al., 2012). This is in line with Grainger and Holcomb’s (2009) proposal that processing up to around 200 ms post-target onset is largely identical for these two types of stimuli mostly involving parallel independent letter processing. Overall, it seems that the VWFA is activated by orthographic stimuli, independent from the lexical status or the pronounceability of the stimuli. In pinpointing a parallel between the present results and the characteristics of the VWFA it is important to observe that several studies reported a marked underactivation of this area in dyslexic individuals (for a review see Richlan et al., 2009).

A recent proposal which helps in placing the letter string deficit shown by children with dyslexia is the dual-route approach to orthographic processing proposed by Grainger and Ziegler (2011). According to this model, the initial mapping of visual features onto abstract letter identities operates in parallel and simultaneously for all the letters in the stimulus (e.g., see also Grainger and van Heuven, 2003; Adelman et al., 2010): “…*the alphabetic array codes for the presence of a given letter at a given location relative to eye fixation along the horizontal meridian. It does not say where a given letter is relative to other letters in the stimulus…. Thus, processing at the level of the alphabetic array is insensitive to orthographic regularity of letter string*” (Grainger and Ziegler, 2011, p. 2). The distinction between non-words, pseudowords, and words would turn out only later in the pathway, when the letter identity is referred to a specific position within the word (defined as a string of letters separated by spaces). Two different types of sublexical word-centerd orthographic representations develop in the reading acquisition process, according to the frequency of occurrence of given combinations of letters: (a) coarse-grained representations (open-bigram representations) that code for the presence of “ordered pairs of letters independently of their contiguity” (e.g., for the string WORD open-bigram representations are WO, WR, WD, OR, OD, RD); (b) fine-grained representations, that code for clusters of frequently co-occurring letter combination (e.g., multi-letter graphemes, syllables, morphemes, rhymes, etc.). The coarse-grained code offers diagnostic features for a rapid bottom-up activation of whole-word representations. However, for the correct identification of the target word is necessary the top-down activation from whole-word orthography level to coarse-grained orthography level. Only real words can activate this interactive process. In the case of pseudowords, the absence of top-down constrains makes the processing via the slower fine-grained route the only way to get disambiguating information on the letter string. Present findings highlight that the global factor explaining dyslexic’s deficit is independent from pronounceability and lexicality of the stimulus. Then, according to the dual-route model (Grainger and Ziegler, 2011), it appears to indicate a deficit at an early stage of processing, i.e., when the initial mapping of visual features onto abstract letter identities is performed. In the subsequent stages of processing, children with dyslexia do not appreciably differ from control readers, as highlighted by the absence of the group by pronounceability or the group by lexicality interactions, once over-additivity was controlled for.

It is interesting to speculate on which mechanism may underlie the selective deficit in processing letter strings shown by children with dyslexia. As stated above, the deficit is confined to the simultaneous processing of several letters while it is much smaller or absent when the task regards single letters or bigrams (e.g., Bosse et al., 2007; Martelli et al., 2009; De Luca et al., 2010) or when the target letters are presented sequentially (Lassus-Sangosse et al., 2008). The present results indicate that children with dyslexia can actually use information from a letter string provided that responding is limited to a single letter presented subsequently to the letter string prime. Thus, the requirements for targets to be multiple and input to be simultaneous seem at the core of the group difference. One reasonable candidate to accommodate for these characteristics is visual crowding. Crowding refers to the decrease in recognizability of a letter surrounded by other letters placed closer than a critical distance (e.g., Pelli et al., 2004, 2007). In the case of letter strings, crowding affects the central letters much more than the initial or final ones (Bouma, 1970); thus, it seems to explain well the single-multiple dimension, as crowding between letters is only expected in the case of multiple displays and not with isolated letters. Further, as a perceptual mechanism, crowding can also easily account for the simultaneity requirement. Early evidence that children with dyslexia show enhanced sensitivity to crowding was presented by Bouma and Legein (1977). In the last years, several studies have shown results compatible with this interpretation (Spinelli et al., 2002; Martelli et al., 2009; Callens et al., 2013; Collis et al., 2013). For example, Martelli et al. (2009) found critical spacing to increase as a function of eccentricity with a greater proportionality for children with dyslexia than typically developing readers. Furthermore, particularly in the dyslexic group, degree of crowding appears to correlate significantly with reading (Martelli et al., 2009; Callens et al., 2013).

It is important to keep in mind that we examined the reading performance of children speaking a very regular language. It is well-known that orthographic consistency modulates the reliance on holistic reading processes (e.g., Ziegler et al., 2001; Ziegler and Goswami, 2006). For this reason, the present findings cannot be directly generalized to inconsistent orthographies, such as English or Hebrew. At any rate, it is interesting that several investigations based on children speaking French, a moderately irregular language, are in keeping with a visual-orthographic, as compared to a visual-to-phonology, impairment (e.g., Lobier et al., 2012a,b; Valdois et al., 2012). For example, Lobier et al. (2012a) reported that, in a visual categorization task with verbal and non-verbal stimuli, children with dyslexia were impaired independently of stimulus type, in keeping with the idea that the impairment was visual and not verbal. These findings suggest that a deficit in pre-lexical graphemic analysis may be present also in inconsistent orthographies, although this possibility certainly deserves further examination.

Overall, children with dyslexia were impaired when they had to process strings, not only of pronounceable stimuli but also of unpronounceable stimuli, a deficit well accounted for by a single global factor. By contrast, they were much less affected when they had to recognize an isolated letter (and no global factor was present) and could take advantage of a pronounceable context, effectively using the ortho-phono-tactic information derived from a previously seen letter string. Therefore, the present findings are in keeping with the proposal that an impairment in pre-lexical graphemic analysis is a core deficit in developmental dyslexia at least in a regular orthography (such as Italian) while they are inconsistent with the alternative view that orthographic–phonological binding may represent a proximal cause of dyslexia.

## Conflict of Interest Statement

The authors declare that the research was conducted in the absence of any commercial or financial relationships that could be construed as a potential conflict of interest.

## Footnotes

1 The exposure time of the multi-element strings was chosen on the basis of a previous pilot study, in which SOAs from 200 to 450 ms were tested. The duration of 350 ms was the time presentation that yielded 75% of accuracy in children with dyslexia.
