# Supplementary material for: Does pronounceability modulate the letter string deficit of children with dyslexia? A study with the rate and amount model
Source: Front Psychol. 2014 Dec 2;5:1353. doi: 10.3389/fpsyg.2014.01353 (PMC4251298; doi:10.3389/fpsyg.2014.01353)
Supplement: Supplementary file 1 [file Data_Sheet_1.PDF]

APPENDIX A: Stimuli used in the lexical decision experimental tasks.

Non-words:

4-letter: NCDR, RNGM, DRGR, CLBD, SPTL, LBDR, SLMB, PLPT, SGMP, RNND, FRBL, TMPS, RLVR, NLSV, NSVR, VRCL;

5-letter: PLLCR, BRLVR, CLDRS, TRSTN, GMLFL, TRRGM, NCRMB, RTRBL, SBDRM, LBDRF, TRPNZ, LNTTM, LMBRC, FRSTN, FLNCL, BLCCR;

6-letter: CRLSTM, DRLMMP, FLNTTL, CRRNGM, LBDSSF, VRGRND, CLCRLP, SLPNNG, BLSTMB, NSPSTN, NSBBRS, STLNZZ, FRLTTM, SNNGMB, NSTTRV, STRNDR.

Pseudowords:

4-letter: BECI, DASU, SAGE, MILU, GAFI, FANI, PULE, URMO, LONI, ERSA, GORE, ZINE, GATI, TENI, LIPA, IRIE;

5-letter: BRADE, FEONO, VAZIE, POTTU, BORRI, SIRSA, ZUBRI, LOSTU, TATTE, MOCCI, VATRI, FROSI, DINNE, FIEMA, PRITE, VONTA;

6-letter: TROGEO, ORNISA, PROGIA, BICCIO, BABITO, MEMMIO, ERTACA, STRIGO, GACCIE, GEBBIO, RAMORO, PORILA, GRIPPE, FARENI, PIOTRA, ASTITE.

Low-frequency words:

4-letter: BACO, DOSE, SUGO, MULO, GUFO, FUNE, PALA, ORMA, NODO, RUGA, DIGA, CUBO, SETA, LIDO, ELMO, PEPE;

5-letter: BRODO, FAUNA, VIZIO, PATTO, BURRO, SORSO, ZEBRA, LISTA, PALCO, BELVA, CEDRO, TROTA, GOLFO, TARGA, NORMA, ATRIO;

6-letter: TREGUA, ARNESE, PREGIO, BUCCIA, BIBITA, MUMMIA, ORTICA, STRAGE, CRESTA, DRAMMA, FLOTTA, CRANIO, ABISSO, VAGONE, CICALA, SAPONE.

High-frequency words:

4-letter: LANA, ORSO, GARA, ZONA, GITA, TANA, LUPO, ARIA, SEME, RANA, ERBA, TOPO, RIVA, NASO, NEVE, VOCE;

5-letter: TETTO, MUCCA, VETRO, FRASE, DONNA, FIUME, PRATO, VENTO, SEDIA, LADRO, TRENO, LATTE, LIBRO, FESTA, FUOCO, BOCCA;

6-letter: GOCCIA, GABBIA, RUMORE, PAROLA, GRUPPO, FARINA, PIETRA, ESTATE, BESTIA, NIPOTE, NEBBIA, STANZA, FRETTA, SANGUE, NATURA, STRADA.
